# Supplementary material for: Influence of COVID-19 on female sex workers in Dar es Salaam, Tanzania: A mixed-methods analysis
Source: PLoS One. 2024 May 31;19(5):e0303993. doi: 10.1371/journal.pone.0303993 (PMC11142675; doi:10.1371/journal.pone.0303993)
Supplement: S1 Table — (DOCX) [file pone.0303993.s001.docx]

Supplemental Table S1: Demographic characteristics of in-depth interview participants

|  | **In-depth interview participants**  **(n=20)** |
| --- | --- |
| **Age** | 29.0 (7.11) |
| **Education** |  |
| None or some primary | 1 (5.0%) |
| Primary complete | 14 (70.0%) |
| Some secondary | 3 (15.0%) |
| Secondary complete | 2 (10.0%) |
| **Marital status** |  |
| Never married/single | 15 (75.0%) |
| Divorced/separated | 4 (20.0%) |
| Married | 0 (0.0%) |
| Widowed | 1 (5.0%) |
| Cohabitating | 0 (0.0%) |
| **Living arrangement** |  |
| Alone | 7 (35.0%) |
| With partner | 0 (0.0%) |
| With family | 10 (50.0%) |
| With friends/other | 3 (15.0%) |
| **Has children** | 18 (90.0%) |
| Data are presented as mean (SD) and frequency (%) | |
