# Supplementary material for: Influence of COVID-19 on female sex workers in Dar es Salaam, Tanzania: A mixed-methods analysis
Source: PLoS One. 2024 May 31;19(5):e0303993. doi: 10.1371/journal.pone.0303993 (PMC11142675; doi:10.1371/journal.pone.0303993)
Supplement: Attachment — Submitted filename: Responsetoreviewers.docx [file pone.0303993.s002.docx]

Many thanks for the very useful comments and suggestions.

Please find below our point-by-point responses (in bold) to each comment.

Best regards,

The authors.

1. When submitting your revision, we need you to address these additional requirements.

Please ensure that your manuscript meets PLOS ONE's style requirements, including those for file naming. The PLOS ONE style templates can be found at 
[https://journals.plos.org/plosone/s/file?id=wjVg/PLOSOne_formatting_sample_main_body.pdf](https://nam11.safelinks.protection.outlook.com/?url=https%3A%2F%2Fjournals.plos.org%2Fplosone%2Fs%2Ffile%3Fid%3DwjVg%2FPLOSOne_formatting_sample_main_body.pdf&data=05%7C02%7Cddewalque%40worldbank.org%7Ca7326d6217b84d9d478008dc47aba153%7C31a2fec0266b4c67b56e2796d8f59c36%7C0%7C0%7C638464050633261322%7CUnknown%7CTWFpbGZsb3d8eyJWIjoiMC4wLjAwMDAiLCJQIjoiV2luMzIiLCJBTiI6Ik1haWwiLCJXVCI6Mn0%3D%7C0%7C%7C%7C&sdata=cbBO9AvsnoVXMd3hh0AazsVHAScRby3Z2sOiBLPjyNQ%3D&reserved=0) and 
[https://journals.plos.org/plosone/s/file?id=ba62/PLOSOne_formatting_sample_title_authors_affiliations.pdf](https://nam11.safelinks.protection.outlook.com/?url=https%3A%2F%2Fjournals.plos.org%2Fplosone%2Fs%2Ffile%3Fid%3Dba62%2FPLOSOne_formatting_sample_title_authors_affiliations.pdf&data=05%7C02%7Cddewalque%40worldbank.org%7Ca7326d6217b84d9d478008dc47aba153%7C31a2fec0266b4c67b56e2796d8f59c36%7C0%7C0%7C638464050633267932%7CUnknown%7CTWFpbGZsb3d8eyJWIjoiMC4wLjAwMDAiLCJQIjoiV2luMzIiLCJBTiI6Ik1haWwiLCJXVCI6Mn0%3D%7C0%7C%7C%7C&sdata=rjNd8RdCc65KbGufY%2BcJ4iqbeVv6tbJsNNGfUg8ytUE%3D&reserved=0).

**Changes made.**

2. Thank you for stating the following financial disclosure: 
 [We are grateful for financial support from the Strategic Impact Evaluation Fund (SIEF) and Knowledge for Change Program (KCP), managed by the World Bank(DdW). These funding sources were not involved in the design or conduct of the research.].  
Please state what role the funders took in the study.  If the funders had no role, please state: ""The funders had no role in study design, data collection and analysis, decision to publish, or preparation of the manuscript."" 
If this statement is not correct you must amend it as needed. 
Please include this amended Role of Funder statement in your cover letter; we will change the online submission form on your behalf.

**This amended role of the funder statement has been added to the manuscript (p. 16) and in the cover letter.**

3. We note that you have indicated that there are restrictions to data sharing for this study. PLOS only allows data to be available upon request if there are legal or ethical restrictions on sharing data publicly. For more information on unacceptable data access restrictions, please see [http://journals.plos.org/plosone/s/data-availability#loc-unacceptable-data-access-restrictions](https://nam11.safelinks.protection.outlook.com/?url=http%3A%2F%2Fjournals.plos.org%2Fplosone%2Fs%2Fdata-availability%23loc-unacceptable-data-access-restrictions&data=05%7C02%7Cddewalque%40worldbank.org%7Ca7326d6217b84d9d478008dc47aba153%7C31a2fec0266b4c67b56e2796d8f59c36%7C0%7C0%7C638464050633275505%7CUnknown%7CTWFpbGZsb3d8eyJWIjoiMC4wLjAwMDAiLCJQIjoiV2luMzIiLCJBTiI6Ik1haWwiLCJXVCI6Mn0%3D%7C0%7C%7C%7C&sdata=CzsAKRMVItlG0Pxx%2FEkdruom240pkU7PDa2MyPH3EUI%3D&reserved=0).

Before we proceed with your manuscript, please address the following prompts:

a) If there are ethical or legal restrictions on sharing a de-identified data set, please explain them in detail (e.g., data contain potentially identifying or sensitive patient information, data are owned by a third-party organization, etc.) and who has imposed them (e.g., a Research Ethics Committee or Institutional Review Board, etc.). Please also provide contact information for a data access committee, ethics committee, or other institutional body to which data requests may be sent.

b) If there are no restrictions, please upload the minimal anonymized data set necessary to replicate your study findings to a stable, public repository and provide us with the relevant URLs, DOIs, or accession numbers. For a list of recommended repositories, please see
[https://journals.plos.org/plosone/s/recommended-repositories](https://nam11.safelinks.protection.outlook.com/?url=https%3A%2F%2Fjournals.plos.org%2Fplosone%2Fs%2Frecommended-repositories&data=05%7C02%7Cddewalque%40worldbank.org%7Ca7326d6217b84d9d478008dc47aba153%7C31a2fec0266b4c67b56e2796d8f59c36%7C0%7C0%7C638464050633283753%7CUnknown%7CTWFpbGZsb3d8eyJWIjoiMC4wLjAwMDAiLCJQIjoiV2luMzIiLCJBTiI6Ik1haWwiLCJXVCI6Mn0%3D%7C0%7C%7C%7C&sdata=tlQWepXTAuIHhXO9zIegmK%2Fm9MiCujlUhMh5jHzYg7E%3D&reserved=0). You also have the option of uploading the data as Supporting Information files, but we would recommend depositing data directly to a data repository if possible.

We will update your Data Availability statement on your behalf to reflect the information you provide.

**We have updated our explanation of the restrictions to sharing the data as follows:**

**Initial explanation:** **Data cannot be shared publicly because of the sensitive nature of the activity (sex work) of the study participants. Data are available from the authors for researchers who meet the criteria for access to confidential data.**

**Revised explanation: Data cannot be shared publicly because of the sensitive nature of the activity (sex work) of the study participants as well as given the sensitive and intimate nature of many of the variables collected (HIV and STI status, sexual behaviors, etc.). The corresponding author (or the World Bank Microdata Library** [**https://microdata.worldbank.org/**](https://microdata.worldbank.org/)**) can be contacted for requests of de-identified data.**

4. Your ethics statement should only appear in the Methods section of your manuscript. If your ethics statement is written in any section besides the Methods, please move it to the Methods section and delete it from any other section. Please ensure that your ethics statement is included in your manuscript, as the ethics statement entered into the online submission form will not be published alongside your manuscript.

**The ethics statement appears in the Methods section (pg. 5).**

Additional Editor Comments:

I commend the authors for this well-written mixed methods paper that addresses shifting dynamics in sex work over the course of the COVID-19 pandemic in urban Tanzania. While I would have preferred to identify two independent peer reviewers to assess the quality of the manuscript, I recognize the review process has already been extensively delayed through fruitless efforts to secure additional reviewers. Thus, I have rendered a decision on the manuscript based on the assessment of one reviewer and myself. In addition to the comments provided by the first reviewer,

I advise the authors to pay particular attention to the following two concerns: (1) Please define how all quantitative measures were captured and assessed in the study methods;

**Response: Details on how all the quantitative measures were captured and assessed have been added to Methods, Data analysis (p. 5):**

**“The following variables were used to describe the sociodemographic characteristics of the study participants and assess their economic security and working landscape pre- and during COVID-19 using univariate analyses (means, standard deviation and frequencies): age, education, marital status, living arrangements, number of children, total monthly income, monthly income from sex work, location of sex work, number of clients, mean price for sex work with clients, and food insecurity.”**

(2) What were drivers of attrition/lost to follow-up reported in wave 3, and were these attributed to the COVID-19 pandemic?

**Response: We have added a more detailed explanation of the possible reasons we experienced wave 3 attrition in the Discussion (p. 14-15)**

**“There was also substantial attrition in wave 3 due to study delays and increased migration related to COVID-19. In addition, new government regulations were introduced which required registration of all mobile phone numbers using biometrics and national ID cards. These regulations resulted in many individuals losing access to their mobile phone numbers, which was our primary mode of contact with study participants. Therefore, it is possible that the balanced sample may be less representative of the overall study sample:”**

I am confident the authors can address these comments expeditiously and improve the quality of the submission. Thank you for the opportunity to review this paper.


[Note: HTML markup is below. Please do not edit.]

Reviewers' comments:

Reviewer's Responses to Questions

**Comments to the Author**

1. Is the manuscript technically sound, and do the data support the conclusions?

The manuscript must describe a technically sound piece of scientific research with data that supports the conclusions. Experiments must have been conducted rigorously, with appropriate controls, replication, and sample sizes. The conclusions must be drawn appropriately based on the data presented.

Reviewer #1: Yes

2. Has the statistical analysis been performed appropriately and rigorously?

Reviewer #1: Yes

3. Have the authors made all data underlying the findings in their manuscript fully available?

The [PLOS Data policy](https://nam11.safelinks.protection.outlook.com/?url=http%3A%2F%2Fwww.plosone.org%2Fstatic%2Fpolicies.action%23sharing&data=05%7C02%7Cddewalque%40worldbank.org%7Ca7326d6217b84d9d478008dc47aba153%7C31a2fec0266b4c67b56e2796d8f59c36%7C0%7C0%7C638464050633290839%7CUnknown%7CTWFpbGZsb3d8eyJWIjoiMC4wLjAwMDAiLCJQIjoiV2luMzIiLCJBTiI6Ik1haWwiLCJXVCI6Mn0%3D%7C0%7C%7C%7C&sdata=pX07DX0CIAG86hC7ZajV2J9sKD%2BQILlprGvzYtuNJtc%3D&reserved=0) requires authors to make all data underlying the findings described in their manuscript fully available without restriction, with rare exception (please refer to the Data Availability Statement in the manuscript PDF file). The data should be provided as part of the manuscript or its supporting information, or deposited to a public repository. For example, in addition to summary statistics, the data points behind means, medians and variance measures should be available. If there are restrictions on publicly sharing data—e.g. participant privacy or use of data from a third party—those must be specified.

Reviewer #1: Yes

4. Is the manuscript presented in an intelligible fashion and written in standard English?

PLOS ONE does not copyedit accepted manuscripts, so the language in submitted articles must be clear, correct, and unambiguous. Any typographical or grammatical errors should be corrected at revision, so please note any specific errors here.

Reviewer #1: Yes

5. Review Comments to the Author

Please use the space provided to explain your answers to the questions above. You may also include additional comments for the author, including concerns about dual publication, research ethics, or publication ethics. (Please upload your review as an attachment if it exceeds 20,000 characters)

Reviewer #1: In this manuscript, the authors used a mixed-methods approach to examine changes in female sex worker’s work environment in the context of changing policies in Tanzania during the COVID-19 pandemic. This paper is well-written and succeeds in capturing shifting dynamics in the sex work landscape during the pandemic. I have just a couple of minor points to offer for the authors to consider.

- Can the authors provide some information on who conducted the interviews and administered the surveys (e.g., gender, credentials, experience/training)?

**Response: Personnel for the field work was entirely composed of females, as all counseling and interviewing should be done with personnel of the same gender as participants. Interviewers and field workers were trained for each specific study procedures: quantitative survey, qualitative interviews and specimen collection. HIV and STI testing, treatment, and all counselling was performed by a partner organization which had qualified and trained staff with several years of experiences in providing HIV/AIDS services to the population in Dar-es-Salam.**

**We have added under the data collection subsection, under methods, p. 4 the fact that all enumerators/interviewers were trained and female. Since this manuscript does not include data from HIV and STI testing, we do not include further details on the qualification of the staff conducting this part of the data collection.**

- How were interview participants sampled (e.g., convenience sampling, other)? Can the authors describe the characteristics of the interview participants?

**Response:** . The female sex workers were recruited using respondent-driven sampling (RDS) within each arm, a chain referral sampling method designed to recruit hard-to-reach and hidden populations. We have further described this method in the study population and design subsection under Methods p. 3. The eligibility criteria for the participants are detailed in the same section and the socio-demographic of the participants are reported in Table 1 and described under results (pp 5-6).

- Can the authors comment on the extent to which they reached data saturation?

**Response: Interviews were conducted until data saturation was reached (i.e., no new information would have been obtained by collecting more data (Morse 1995)).** **This information has been added under Data collection, p. 4.**

6. PLOS authors have the option to publish the peer review history of their article ([what does this mean?](https://nam11.safelinks.protection.outlook.com/?url=https%3A%2F%2Fjournals.plos.org%2Fplosone%2Fs%2Feditorial-and-peer-review-process%23loc-peer-review-history&data=05%7C02%7Cddewalque%40worldbank.org%7Ca7326d6217b84d9d478008dc47aba153%7C31a2fec0266b4c67b56e2796d8f59c36%7C0%7C0%7C638464050633297719%7CUnknown%7CTWFpbGZsb3d8eyJWIjoiMC4wLjAwMDAiLCJQIjoiV2luMzIiLCJBTiI6Ik1haWwiLCJXVCI6Mn0%3D%7C0%7C%7C%7C&sdata=hHkwW02qIS9Nv1bY2Cum6ofIMrC5hZnCkeuOQjIrsLE%3D&reserved=0)). If published, this will include your full peer review and any attached files.


If you choose “no”, your identity will remain anonymous but your review may still be made public.


**Do you want your identity to be public for this peer review?** For information about this choice, including consent withdrawal, please see our [Privacy Policy](https://nam11.safelinks.protection.outlook.com/?url=https%3A%2F%2Fwww.plos.org%2Fprivacy-policy&data=05%7C02%7Cddewalque%40worldbank.org%7Ca7326d6217b84d9d478008dc47aba153%7C31a2fec0266b4c67b56e2796d8f59c36%7C0%7C0%7C638464050633306190%7CUnknown%7CTWFpbGZsb3d8eyJWIjoiMC4wLjAwMDAiLCJQIjoiV2luMzIiLCJBTiI6Ik1haWwiLCJXVCI6Mn0%3D%7C0%7C%7C%7C&sdata=7qKVwDVWur67zEG0pwH7Vdgu3dO7l0irif%2B%2FvFtVOd8%3D&reserved=0).

Reviewer #1: No
